# Recurrent Submandibular Sialadenitis With Associated Submandibular Lymphadenopathy Secondary to Iodinated Contrast: A Case Report

**DOI:** 10.7759/cureus.107802

**Published:** 2026-04-27

**Authors:** Naishal Mandal, Majd Hasan, Shaza Mohammed, Omer Kamal Ahmed Elobeid, Adiraj Singh

**Affiliations:** 1 Internal Medicine, Hurley Medical Center/Michigan State University (MSU) College of Human Medicine, Flint, USA; 2 Internal Medicine and Pediatrics, Hurley Medical Center/Michigan State University (MSU) College of Human Medicine, Flint, USA

**Keywords:** cervical lymphadenopathy, contrast-induced sialadenitis, iodide mumps, iodinated contrast, recurrent contrast reaction, salivary gland edema, self-limited reaction, submandibular swelling

## Abstract

Iodinated contrast media are essential diagnostic tools widely used in radiographic and computed tomography (CT) examinations, with millions of procedures performed annually. Although generally well tolerated, these agents can, rarely, cause adverse reactions, including hypersensitivity reactions, acute kidney injury, and contrast-induced sialadenitis (iodide mumps). Contrast-induced sialadenitis is an uncommon, benign reaction to iodinated contrast media that most frequently involves the parotid glands and is likely underrecognized. Submandibular gland involvement with associated cervical lymphadenopathy is particularly rare and may mimic infectious or obstructive salivary gland pathology.

We report a case of a 64-year-old woman who developed acute bilateral submandibular swelling and pain three to four hours after iodinated contrast administration for a CT performed to evaluate acute abdominal pain. The patient had a history of similar, self-limited episodes following prior contrast exposure. Physical examination revealed diffuse submental and submandibular swelling with mild tenderness but no signs of infection or airway compromise. Ultrasound demonstrated bilateral submandibular gland enlargement with heterogeneous echotexture, increased vascularity, and reactive cervical lymphadenopathy. Based on the temporal relationship to contrast exposure, prior similar episodes, absence of infectious features, and supportive imaging findings, a diagnosis of contrast-induced submandibular sialadenitis was made. Symptoms improved rapidly with conservative management and resolved within two days.

This case highlights the importance of recognizing contrast-induced submandibular sialadenitis as a rare but benign and self-limiting condition. Awareness of this entity can help clinicians avoid unnecessary antibiotics, corticosteroids, and extensive diagnostic workup. Proper recognition and documentation are also important for counseling patients about future contrast exposure and considering alternative imaging modalities when appropriate.

## Introduction

Iodinated contrast media are routinely used in diagnostic and interventional imaging and are generally well tolerated [1]. Contrast-induced sialadenitis is a rare adverse reaction characterized by acute, painless or mildly painful swelling of one or more salivary glands, occurring minutes to several days after contrast exposure, and is typically self-limiting [1,2]. Although parotid gland involvement is most common, submandibular gland involvement is uncommon and has been described primarily in individual case reports and small case series [3-5].

Contrast-induced sialadenitis is thought to occur due to iodide accumulation within salivary gland tissue through active transport mechanisms, leading to glandular edema and inflammation [1,6]. Iodinated contrast media are classified based on their chemical structure and osmolality into ionic, high-osmolar, and non-ionic low-osmolar contrast media [7,8]. Non-ionic contrast agents, such as iopamidol, are associated with a lower incidence of immediate hypersensitivity reactions compared with ionic preparations [7,8].

Adverse effects of iodinated contrast media are typically mild and self-limited, including nausea, vomiting, flushing, and urticaria, while more severe reactions, such as anaphylactoid responses, are uncommon [8]. Most reactions occur shortly after administration and are dose-independent, whereas delayed reactions are less frequent and usually cutaneous in nature [8]. Recognition of this rare entity is important to distinguish it from infectious sialadenitis, obstructive pathology, or IgE-mediated hypersensitivity reactions [8].

We report a case of post-contrast submandibular sialadenitis with associated submandibular lymphadenopathy in a patient undergoing evaluation for acute abdominal pain.

## Case presentation

A 64-year-old woman with a past medical history of hypertension and gastroesophageal reflux disease (GERD) presented to the Emergency Department at Hurley Medical Center in Flint, MI, USA, with acute lower abdominal pain, nausea, and decreased appetite. She reported progressively worsening abdominal pain over seven days, described as constant, dull, and aching, rated 5/10 in intensity, localized to the left lower quadrant, with radiation toward the suprapubic region and rectum. The pain was aggravated by bowel movements, oral intake, and movement, and was partially relieved by rest. She also reported constipation and abdominal bloating but denied hematochezia, melena, fever, chills, diarrhea, vomiting, dysphagia, urinary symptoms, or respiratory distress. She had no prior similar abdominal symptoms.

On physical examination, the patient was hemodynamically stable. Abdominal examination revealed localized tenderness in the left lower quadrant and suprapubic region, with mild voluntary guarding but no rebound tenderness or rigidity. Bowel sounds were present and normoactive. There was no abdominal distention, palpable mass, or costovertebral angle tenderness.

Initial laboratory evaluation demonstrated mild leukocytosis with a white blood cell (WBC) count of 12.1 × 10³/µL. Lipase, liver function tests, and the basic metabolic panel were within normal limits (Table 1).

**Table 1:** Initial laboratory work-up

Laboratory Work-Up	Patient Value	Reference Range (Normal)
White Blood Cell Count	12.1 × 10³/µL	4.0 - 11.0 × 10³/µL
Hemoglobin (HGB)	13.6 g/dL	Male: 13.5 - 17.5 g/dL; Female: 12.0 - 15.5 g/dL
Platelet Count	263 × 10³/µL	150 - 450 × 10³/µL
Neutrophill	72%	40 - 70%
Eosinophill	1%	0 - 6%
Liver Function Tests (LFTs)		
AST (Aspartate Aminotransferase)	26 U/L	10 - 40 U/L
ALT (Alanine Aminotransferase)	27 U/L	7 - 56 U/L
ALP (Alkaline Phosphatase)	100 U/L	44 - 147 U/L
Total Bilirubin	0.9 mg/dL	0.1 - 1.2 mg/dL
Pancreatic Enzyme		
Lipase	30 U/L	0 - 160 U/L

**Figure 1:**
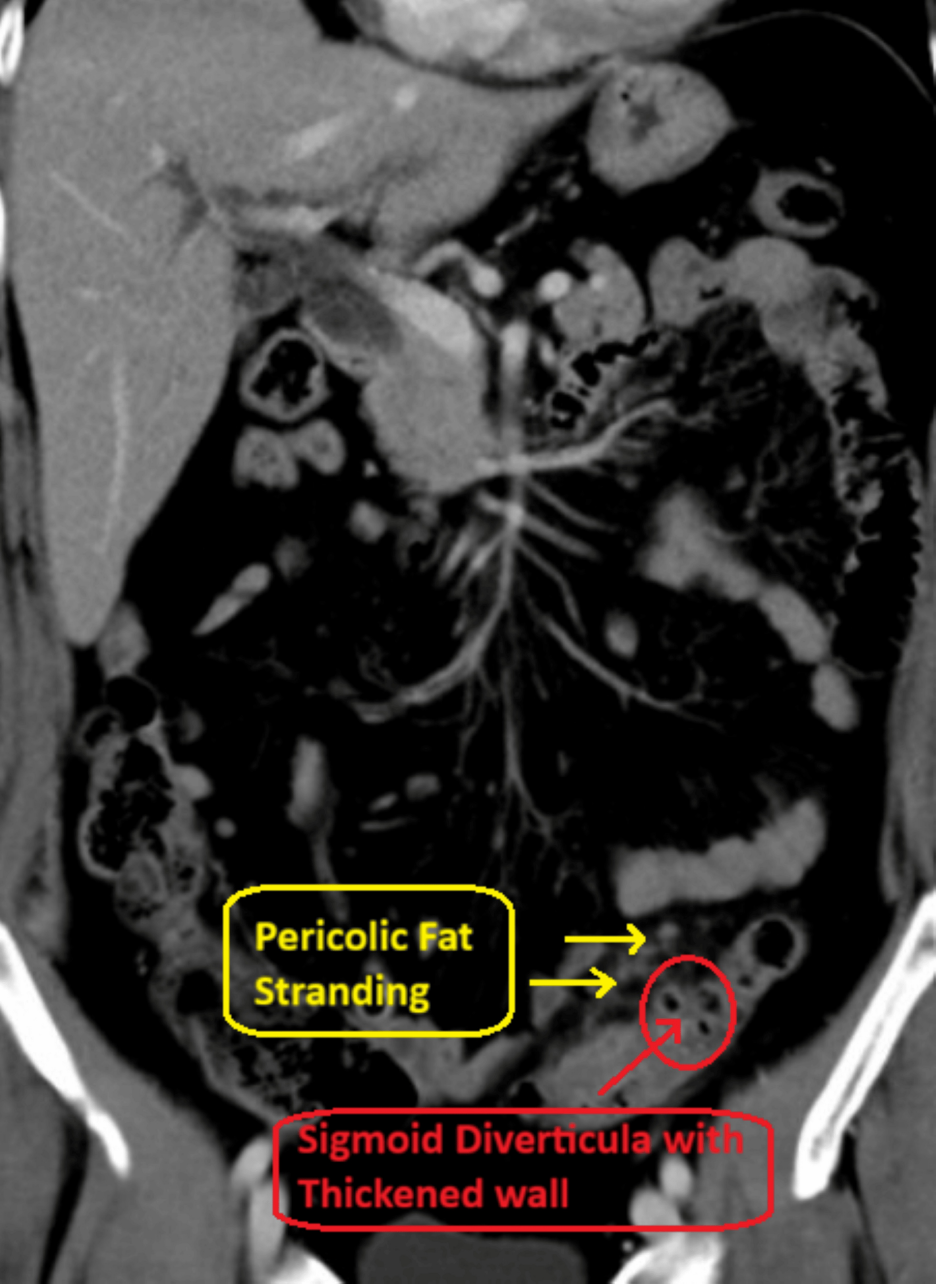
Contrast-enhanced computed tomography (CT) scan of the abdomen and pelvis Contrast-enhanced computed tomography (CT) scan of the abdomen and pelvis with IV contrast (coronal view) demonstrating sigmoid diverticulitis, characterized by the presence of sigmoid diverticula with associated colonic wall thickening and surrounding pericolic fat stranding, without evidence of abscess, perforation, or fistula formation.

Given the persistent localized pain and leukocytosis, a contrast-enhanced computed tomography (CT) scan of the abdomen and pelvis was performed. The patient received intravenous iodinated contrast (Omnipaque 300, 100 mL), as well as oral contrast (Omnipaque 300, 25 mL diluted in 500 mL of water, administered two hours prior to imaging and repeated one hour before the study). CT imaging demonstrated sigmoid diverticulitis, characterized by the presence of sigmoid diverticula with associated colonic wall thickening and surrounding pericolic fat stranding, without evidence of abscess, perforation, or fistula formation (Figure 1).

Approximately three to four hours after receiving iodinated contrast, the patient developed acute swelling and dull pain beneath the chin. Physical examination revealed diffuse submental and submandibular swelling, with mild tenderness. There was no overlying erythema, airway compromise, dysphagia, or respiratory distress. Oral examination showed no swelling or erythema of the floor of the mouth, and dentition was normal. The patient reported two prior similar episodes of neck swelling following iodinated contrast exposure, both of which resolved spontaneously within five days without treatment.

**Figure 2:**
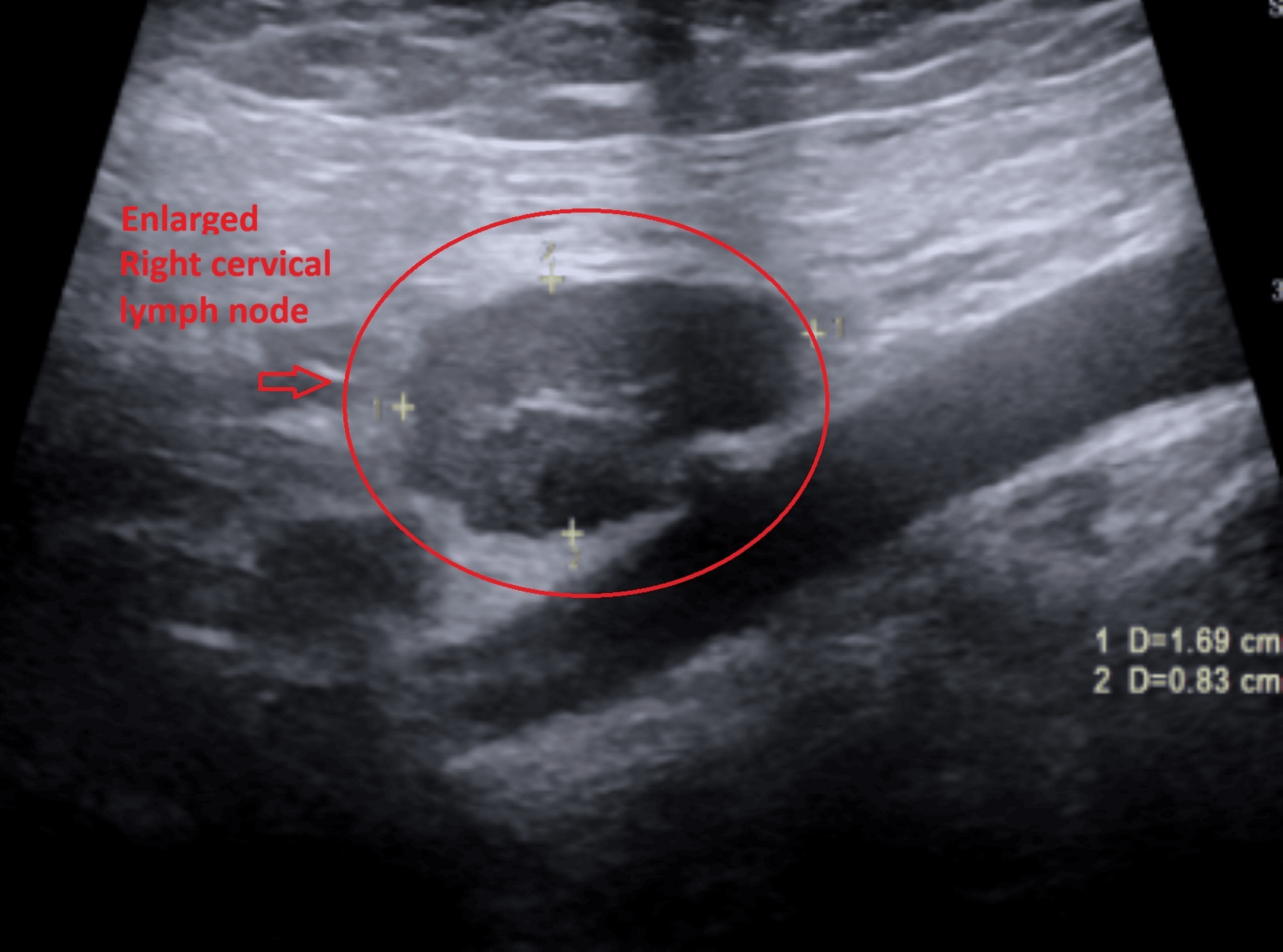
Ultrasound image of the largest right cervical lymph node in the sagittal view

**Figure 3:**
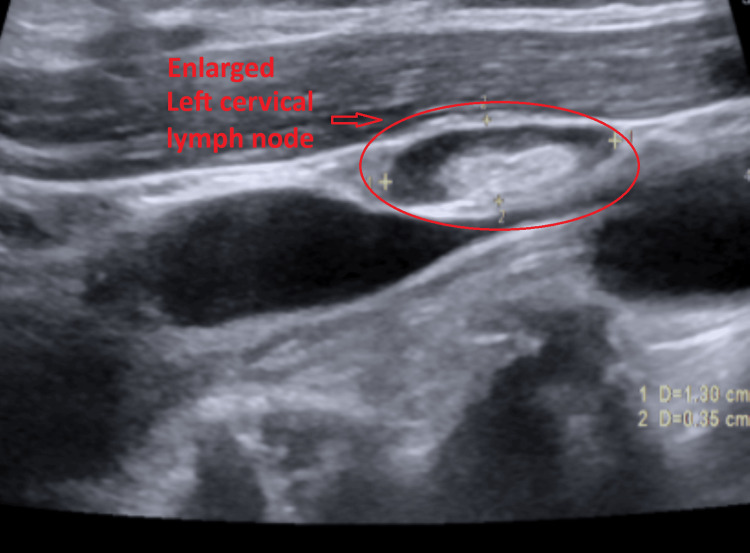
Ultrasound image of the largest left cervical lymph node

**Figure 4:**
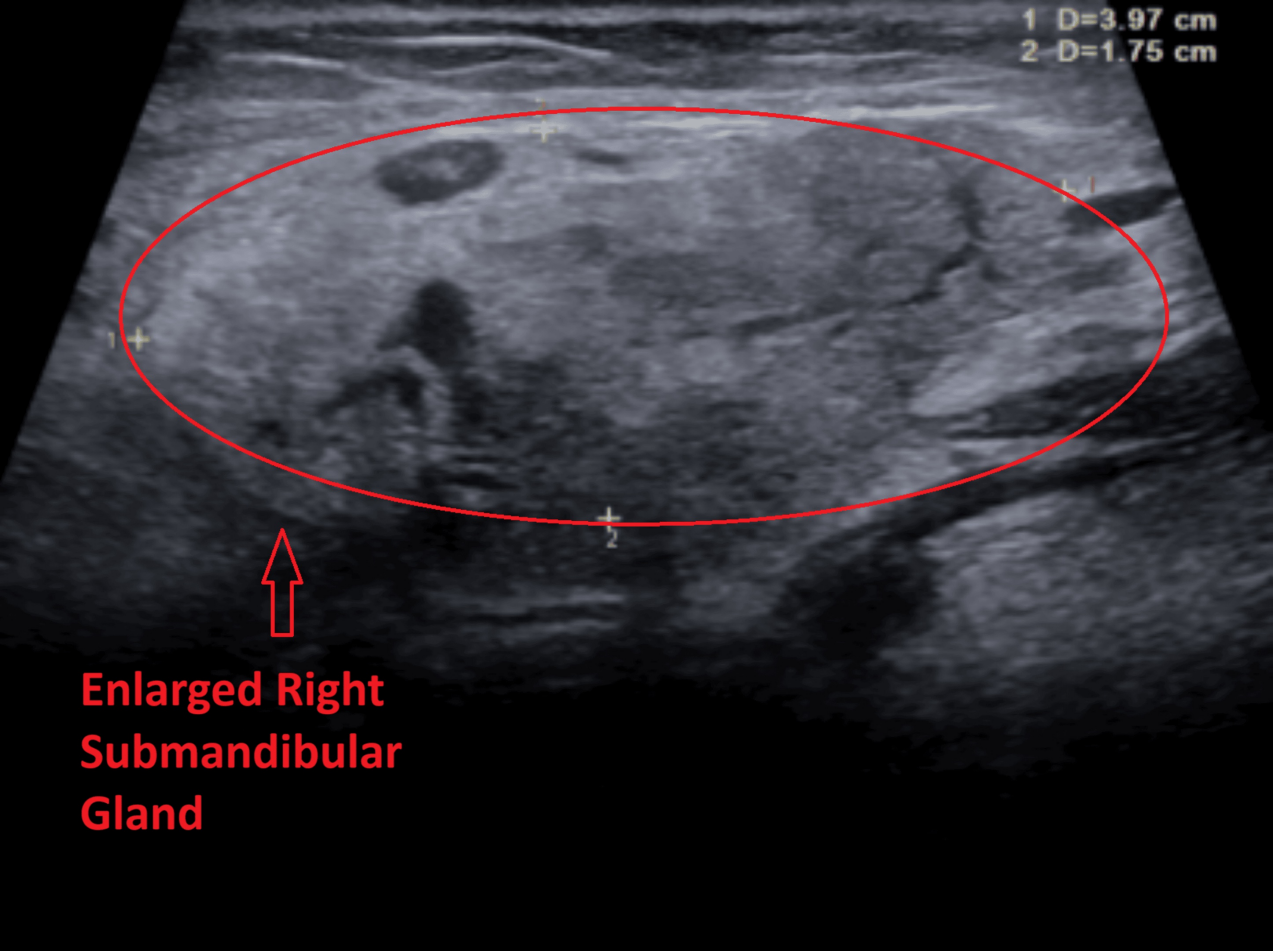
Ultrasound image of the transverse view of the right submandibular gland

**Figure 5:**
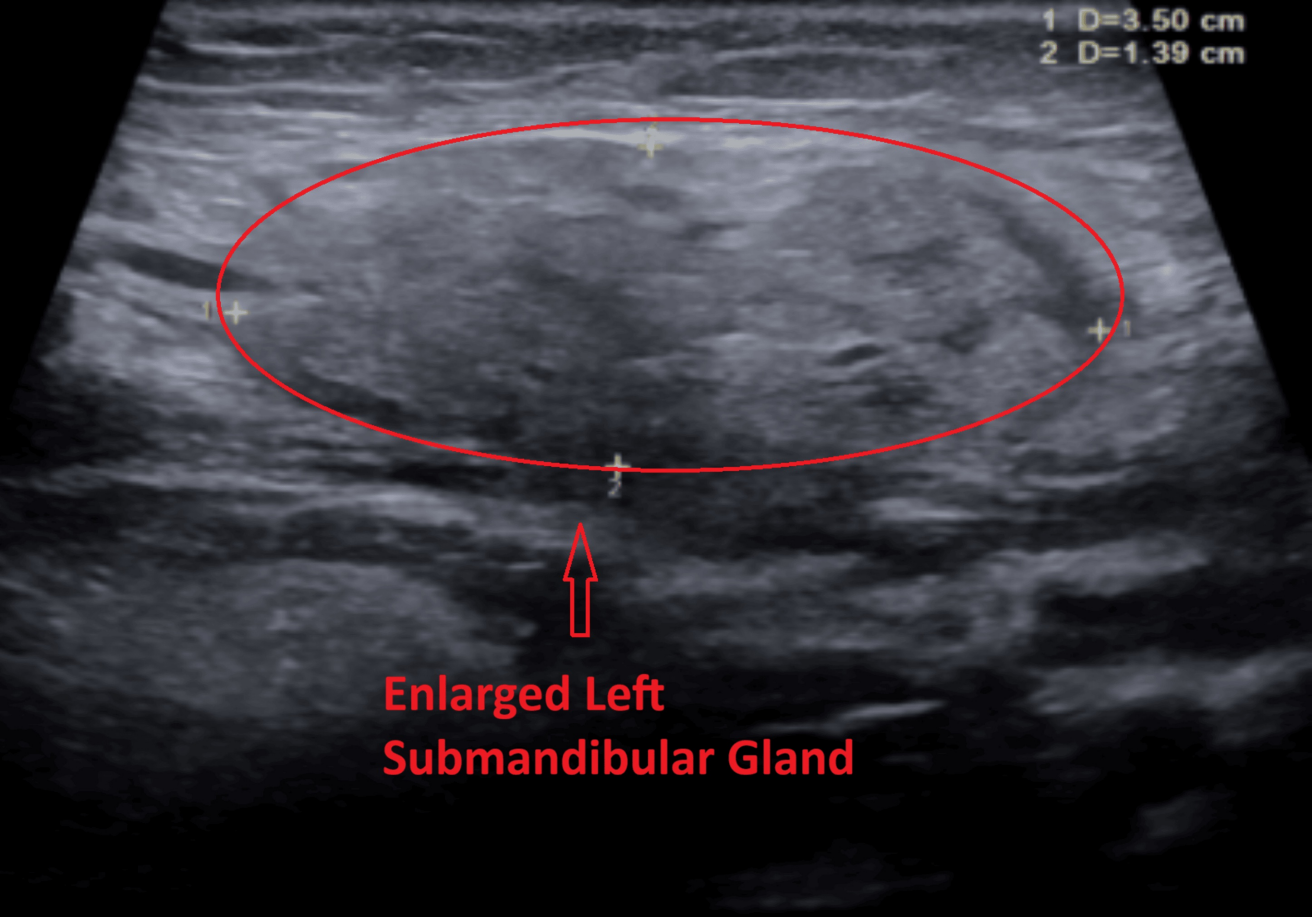
Ultrasound image of the transverse view of the left submandibular gland

**Figure 6:**
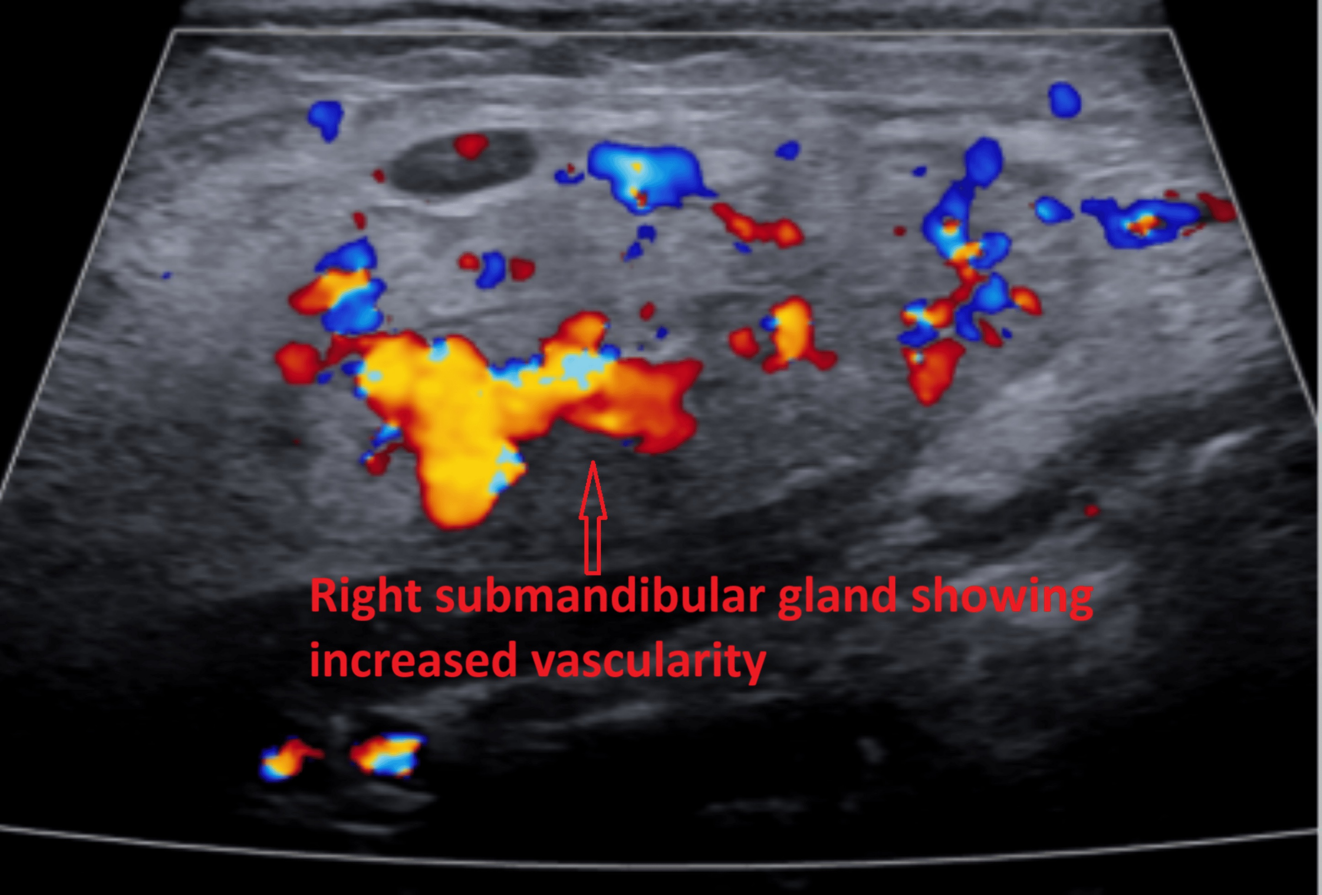
Color Doppler ultrasound image of the transverse view of the right submandibular gland showing increased vascularity

**Figure 7:**
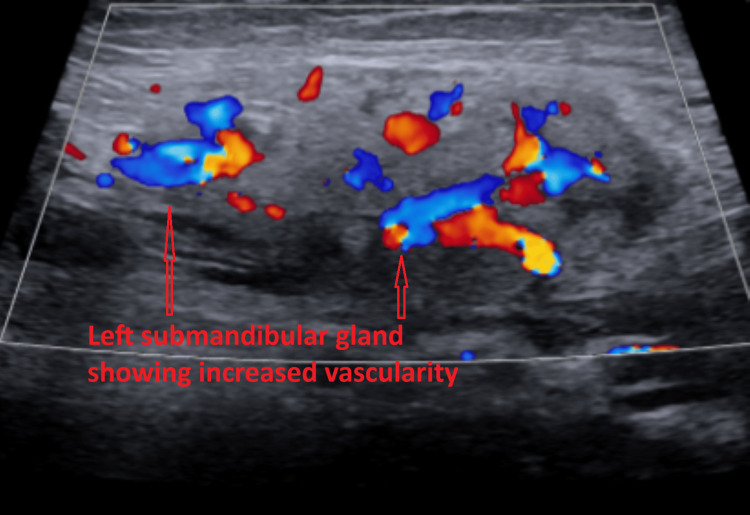
Color Doppler ultrasound image of the transverse view of the left submandibular gland showing increased vascularity

Subsequent ultrasound of the soft tissues of the neck demonstrated multiple bilateral hypoechoic cervical lymph nodes with central echogenic hila, consistent with reactive lymphadenopathy. The largest lymph node measured 1.7 × 0.8 × 1.2 cm on the right (Figure 2) and 1.3 × 0.4 × 0.8 cm on the left (Figure 3). No fluid collection or abscess was identified. Additionally, both submandibular glands were mildly enlarged with heterogeneous echotexture (Figures 4-5) and mildly increased vascularity (Figures 6-7), findings suggestive of an inflammatory process.

The diagnosis of contrast-induced submandibular sialadenitis with reactive lymphadenopathy was made based on the characteristic temporal relationship to iodinated contrast exposure, recurrence of similar episodes after prior contrast administration, absence of clinical features suggestive of infection, and supportive ultrasound findings demonstrating gland enlargement without abscess formation.

Given the benign and self-limited nature of this condition, conservative management was chosen. No antibiotics, corticosteroids, or invasive interventions were initiated. The submandibular swelling gradually improved and significantly decreased within two days. The patient remained clinically stable and was discharged with reassurance and outpatient follow-up. No complications were observed.

## Discussion

Contrast-induced sialadenitis, commonly termed iodide mumps, is an uncommon adverse reaction that may occur after exposure to iodinated contrast agents [1,2]. It has been reported to develop within minutes to several days after contrast administration and appears independent of dose and route of delivery [2]. Ultrasound imaging typically reveals diffuse glandular enlargement without abscess formation or suppurative features, aiding differentiation from infectious sialadenitis [1,2]. Both ionic and non-ionic iodinated contrast agents have been implicated in published case reports and reviews [1,3].

The proposed pathophysiologic mechanism is mediated by the sodium-iodide symporter (NIS), an intrinsic transmembrane glycoprotein that facilitates active iodide transport into epithelial cells through coupling with the sodium gradient generated by Na⁺/K⁺-ATPase activity [4]. Although NIS expression is most prominent in thyroid follicular cells, it is also physiologically expressed in extrathyroidal tissues, including the salivary glands, gastric mucosa, and lactating mammary glands [5]. Within salivary glands, NIS is localized predominantly to the basolateral membrane of ductal and acinar epithelial cells, enabling iodide concentration within glandular tissue [5,6].

Following administration of iodinated contrast, circulating iodide concentrations increase acutely, resulting in enhanced NIS-mediated iodide uptake by salivary gland epithelium [6]. Unlike the thyroid gland, salivary tissue lacks efficient iodide organification mechanisms, which may allow transient intracellular and intraductal accumulation of iodide [5,7]. Elevated intraglandular iodide concentrations may increase osmotic load, promote interstitial edema, and induce ductal obstruction with secondary inflammatory changes [3,7]. Reactive cervical lymphadenopathy has been described in association with contrast-induced sialadenitis and is considered an inflammatory response rather than evidence of infection [2,8].

Renal function may influence susceptibility, as impaired renal clearance can prolong systemic iodide exposure and increase salivary gland accumulation [8,9]. Nevertheless, cases have also been described in patients with normal renal function, suggesting that individual glandular susceptibility and contrast volume may contribute to pathogenesis [1,8]. In neuroendovascular cohorts, higher contrast volumes and specific catheter positioning have been associated with increased risk [9].

Submandibular gland involvement is less common than parotid gland involvement but has been documented in case reports and series [10]. The differential diagnosis includes acute bacterial sialadenitis, sialolithiasis, angioedema, autoimmune sialadenitis, and neoplastic processes [8,10].

The incidence of contrast-induced sialadenitis is likely underrecognized, with reported estimates ranging from approximately 1% to 4.2% in selected procedural cohorts [8,9]. Management is primarily supportive and includes observation, hydration, and analgesia, while corticosteroids or antihistamines may be used selectively in symptomatic cases [1,8]. The overall prognosis is excellent, with complete resolution expected in the majority of patients [1,8,11].

Multiple case reports confirm the benign and self-limited nature of iodide mumps following contrast-enhanced imaging studies [11,12].

**Table 2:** Summary of published reports on contrast-induced sialadenitis and related NIS studies Meta-analysis and cohort studies report aggregated demographic and clinical data rather than individual case-level data. "Time to self-resolution" reflects reported clinical recovery without invasive intervention. NIS: Sodium-Iodide Symporter; N/A: Not Applicable (basic science or mechanistic study without patient-level clinical data); B/L: Bilateral

Published Reports and Related NIS Studies	Age (years)	Sex	Major Salivary Gland Affected	Time of Onset After Iodine Exposure	Time to Self-Resolution
Lucarelli et al. [1]	73	Female	Parotid (B/L)	~12 hours	3-5 days
Federici et al. [2]	76	Male	Submandibular (B/L)	<24 hours	2-3 days
Zhang et al. [3]	65	Female	Submandibular	Few hours	2 days
Dai et al. [4]	N/A	N/A	N/A (NIS molecular study)	N/A	N/A
Spitzweg et al. [5]	N/A	N/A	N/A (NIS expression study)	N/A	N/A
La Perle et al. [6]	N/A	N/A	N/A (NIS salivary modulation study)	N/A	N/A
Mandel SJ and Mandel L [7]	N/A	N/A	Parotid/Submandibular	Variable	Variable
Jiao et al. [8] (Meta-analysis)	~61	Male/Female	Parotid > Submandibular	Minutes to several days	1-2 weeks (Majority)
Lee et al. [9] (Cohort)	~63	Male/Female	Parotid predominant	Within 24 hours	Several days
Pennacchio et al. [10]	58	Female	Submandibular	24 hours	5 days
Gilgen-Anner et al. [11]	52	Female	Parotid (B/L)	1 day	7 days
Chow et al. [12]	68	Female	Parotid	Within hours	3 days
Panasoff and Nusem [13]	62	Male	Parotid	24-48 hours	Several days
Wylie and Mitchell [14]	59	Female	Parotid	Within 24 hours	4-7 days

This case report has several limitations. This report describes a single patient, which limits the generalizability of the findings. The diagnosis was made clinically based on temporal association, prior similar episodes, and supportive ultrasound findings; no histopathologic confirmation was obtained. Although infectious and obstructive causes were considered unlikely based on clinical and imaging features, complete exclusion of alternative etiologies cannot be definitively established. Additionally, the exact type and volume of iodinated contrast agent were not analyzed in relation to symptom severity, and long-term follow-up data were limited. Larger studies are needed to better define the true incidence, pathophysiology, risk factors, recurrence rates, and optimal preventive strategies for contrast-induced submandibular sialadenitis.

## Conclusions

This case describes post-contrast submandibular sialadenitis with associated lymphadenopathy occurring after iodinated contrast exposure, a rare but benign and self-limited reaction. In this patient, the diagnosis was supported by the clear temporal relationship to contrast administration, recurrence after prior exposures, characteristic imaging findings, and rapid clinical improvement without specific treatment. Although conclusions from a single case should be interpreted cautiously, this report highlights the importance of recognizing contrast-induced sialadenitis as a potential cause of acute salivary gland swelling following contrast administration. Increased clinical awareness may help clinicians distinguish this entity from infectious or obstructive causes, thereby avoiding unnecessary diagnostic investigations, antibiotic therapy, or invasive interventions.
